# Histomorphometric and biochemical data of rat kidney submitted to warm ischemia associated with resveratrol treatment

**DOI:** 10.1016/j.dib.2020.105545

**Published:** 2020-04-15

**Authors:** Gabriela F. Buys-Gonçalves, Francisco J.B. Sampaio, Marco A. Pereira-Sampaio, Maria Eduarda M. Silva, Diogo B. De Souza

**Affiliations:** a Urogenital Research Unit, State University of Rio de Janeiro, Rio de Janeiro, Brazil; b Department of Morphology, Fluminense Federal University, Niteroi, Brazil; c Department of Veterinary Medicine, Educational Foundation Serra dos Órgãos, Teresópolis, Brazil

**Keywords:** Rat kidney, Warm ischemia, Resveratrol, Histomorphometry

## Abstract

The data presented here come from the article "Histomorphometric evaluation of the rat kidney submitted to warm ischemia and the protective effect of resveratrol" [1]. Rats of Wistar lineage (*n* = 39; 9 weeks of age) were obtained and apportioned into 4 groups at random. Both groups Sham (S) and Sham Resveratrol (SR) were submitted to open laparotomy and dissection of the left renal pedicle, the same as groups Ischemia (I) and Ischemia Resveratrol (IR), being the last two also submitted to 1 h left warm renal ischemia. SR and IR were treated with 30 mg/kg of resveratrol intraperitoneally 1 h before the surgical procedure, while S and I received saline injections. Rats were killed a month after surgery by anesthetic overdose. A blood sample was collected by cardiac puncture for determination of serum urea and creatinine serum by biochemical analysis at automated enzymatic method. Kidneys were weighted, Sherle´s method was used for measurement of their volume and then both were fixated in buffered formalin for 48 h. Cortex-non-cortex areas ratio (C—NC) was assessed by Cavalieri's method using a stereoscope. The product of multiplying the renal volume by the C—NC is the cortical volume (CV). Left kidneys fragments were processed for histology resulting in slides that were stained with haematoxylin and eosin. For histomorphometric analyses, 25 random cortical fields were photographed at 200x magnification using a camera attached to a light microscope. The estimation of glomerular volumetric density (Vv [Glom]), indication of proportional volume occupied by glomeruli in the cortex, was performed by the point-counting method. The point-sampled intercepts method was used to estimate the volume-weighted mean glomerular volume (VWGV). Total number of glomeruli per kidney (N [Glom]) estimation was achieved through the formula CVxVv [Glom]/VWGV. All the data were tabulated in spreadsheets. The quantitative results were compared by one-way ANOVA with Tukey's post-test using GraphPad Prism software. All results were considered significant when the value of *p* <0.05.

Specifications table

**Table:** 

Subject	Surgery
Specific subject area	Renal cancer surgery. Partial nephrectomy. Renal warm ischemia.
Type of data	Table
	Figure
	Graph
How data were acquired	Biochemistry kits: urea (Urea UV, REF 104–4 / 50, Lote 7013, Labtest, Lagoa Santa, Brazil) and creatinine (Creatinina K, REF 96–300, Lote 7013, Labtest). Apparatus: semiautomatic biochemical analyser (Bioplus BIO-2000, Barueri, Brazil), precision scale (Marte AD2000, São Paulo, Brazil), Axiocam 506 color (Carl Zeiss Microscopy, LLC, Jena, Germany) attached to the Stereo Discovery.V8 stereoscope (Carl Zeiss) and light microscope (Olympus BX51, Tokyo, Japan) equipped with a digital camera (Olympus DP71, Tokyo, Japan).
	Softwares: Zaiss Axiocam for Windows, Olympus Cam for Windows, Microsoft Excel 2016, Image J for Windows and GraphPad Prism 8.3.1 for Windows.
Data format	Raw
	Analysed
Parameters for data collection	The Wistar rats used were all male, clinically healthy, with an average weight of 320 g and 9 weeks of age. They were kept in polypropylene boxes with a maximum number of 3 animals/box, in a vivarium environment with light/dark cycle and were offered water and commercial feed ad libitum. They were divided into four groups at random. The treatment of animals in the Untreated (S and I) and Treated (SR and IR) groups was conducted in a standardized manner, and the same can be said for surgical procedures, euthanasia, blood collection and analysis of urea and creatinine, dissection and kidney collection, weighing, volume measurement, cleavage, buffered formalin fixation, Cavalieri method slice analysis for the cortex-non-cortex areas ratio (C—NC), histological processing, histological slide making and stain, drying, random photomicrographs, glomerular volumetric density (Vv [Glom]) and volume-weighted mean glomerular volume (VWGV) analysis, data tabulation and statistical analysis. P-value was always considered significant when <0.05.
Description of data collection	During each animal's anesthetic plan, a cardiac puncture was performed to collect blood, which was subsequently centrifuged to obtain the serum and thus, the serum urea and creatinine dosage by a semiautomatic biochemical analyser. The abdominal cavity was then opened to access the kidneys, which were dissected, collected and weighed. The renal volume was measured by the Scherle's method, which is used to determine the volume of bodies with an
	irregular surface based on the Archimedes principle, that is, a body totally or partially immersed in a fluid undergoes a thrust that is equal to the weight of the volume of the fluid displaced by the body. To measure it, the weight (W) is recorded to be given by the displacement of an isotonic saline solution by the organ volume. As the density (σ) of the isotonic saline solution is 1.0048 and the volume (V) is obtained by the formula: *V* = *W*/σ, the volume value is like the weight (*V* ≈ *W*). The kidneys were cleaved transversely in the hilar region and fixed in separate flasks containing 3.7% buffered formalin solution. Isotropic, uniform and random fragments of the kidneys were obtained using the vertical cleavage method. The latter were routinely processed for histology in a processor with dehydration by ethyl alcohol baths in increasing concentrations, followed by clarification in xylol and, finally, embedded and embedded in paraffin in the apparatus. After slicing 5 μm thick slices through the microtomy and making slides. After drying, the slides were stained by the haematoxylin and eosin method for histopathological and stereological Vv[Glom] and VWGV. The proportional area of the cortex and non-cortex regions (medulla, capsule and adipose tissue of the renal sinus) was calculated using Cavalieri's method using the ImageJ software. The kidneys were sectioned into seven to eight transverse slices 2 mm thick and a transvesal surface of each slice was photographed in a camera attached to the stereomicroscope, along with a ruler millimeter for further calibration of ImageJ. The images were analysed under 15x magnification. First, the distance occupied by a specified number of pixels of the image in millimeters is calibrated using the ruler. After calibration, the total area of the slice and the area of the non-cortical region in each image were firstly analysed using the “Polygon selections” tool and, through subtraction, it was possible to calculate the area of the cortical region. Multiplying the value obtained by the volume obtained by the Scherle method, it was possible to calculate the volume of each region, cortical (CV) and non-cortical. Vv [Glom] is given by Pp/Pt, where Pp is the number of points that overlap the glomeruli and Pt is the total number of points in the grid (42). The Vv is given in percentage and, multiplying this value by the VC and dividing the result by 100, the numerical value of the absolute glomerular density is found in milliliters. The VWGV was estimated using the point interception method. Over the length of the glomerulus intercepted by the line of a grid with lines parallel to each other, a logarithmic ruler of 32 mm in length is placed, composed of a series of 15 classes. This grid is placed over the image at randomly selected angles, with the angle being recalculated, at random, for each image analysed (ranging from 5° to 90°, with 5° intervals). The number of glomeruli per cubic millimeter of renal cortex (N [Glom]) was calculated using the formula VCxVv [Glom]/VWGV.
Data source location	Institution: State University of Rio de Janeiro
	City/Town/Region: Rio de Janeiro
	Country: Brazil
Data accessibility	With the article
Related research article	Buys-Gonçalves GF, Sampaio FJB, Silva MEM, Pereira-Sampaio MA, De Souza DB. Histomorphometric evaluation of the rat kidney submitted to warm ischemia and the protective effect of resveratrol. Am J Surg. 2020; doi:10.1016/j.amjsurg.2020.02. In press.

## Value of the data

• These data bring positive parameters and evidence regarding the nephroprotective effect of resveratrol related to warm ischemia in Wistar rats, an experimental model widely used in preclinical trials
• Such data may be beneficial for researchers who wish to justify studies involving the protective effects of resveratrol against renal or oxidative ischemic damage more generally. Thus, urologists and nephrologists who wish to research and/or use this bioflavonoid as a complementary treatment for their patients who will undergo partial nephrectomy can also benefit
• Scholars with lines of research involving bioflavonoids, or specifically resveratrol, can use this data and methodology to carry out related research, since they can be used as a complement and reference
• The present data demonstrate that resveratrol protects the kidney against damage from warm ischemia in a quantitative, that is, absolute way. This is because the number of glomeruli per kidney is very close to the number of remaining nephrons

## 1 Data description

**Fig. 1:**
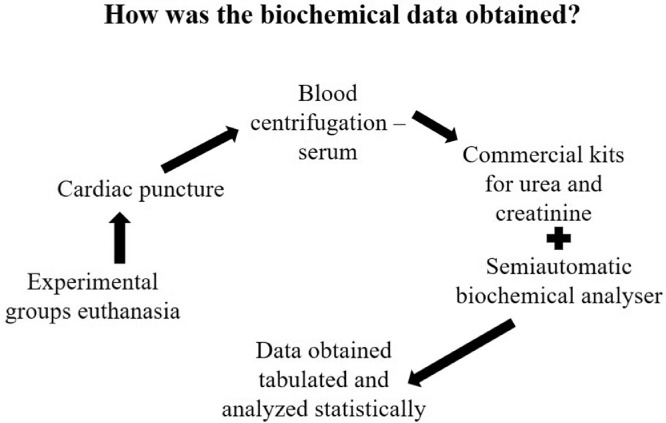
How was the biochemical data obtained?.

**Fig. 2:**
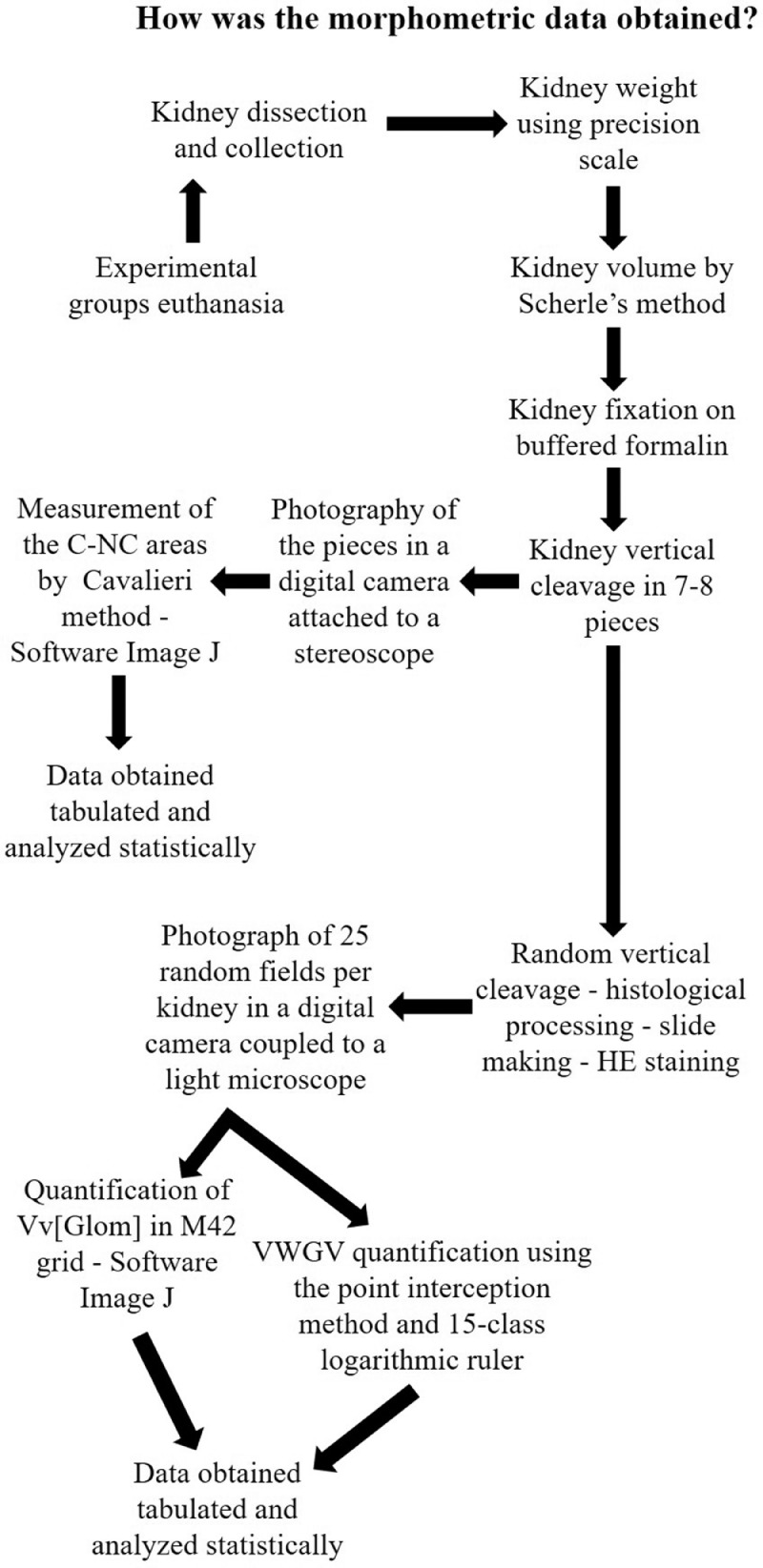
How was the morphometric data obtained?.

**Fig. 3:**
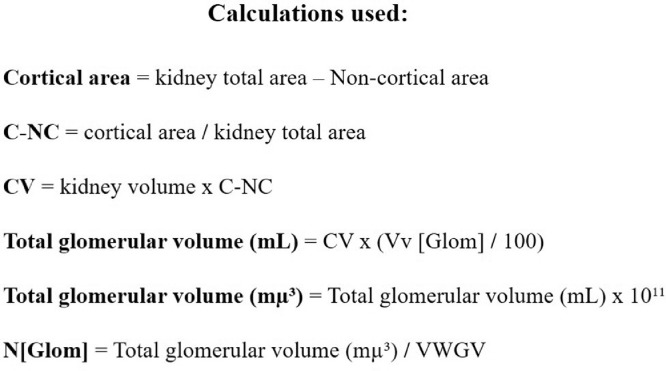
Calculations Used.

**Fig. 4:**
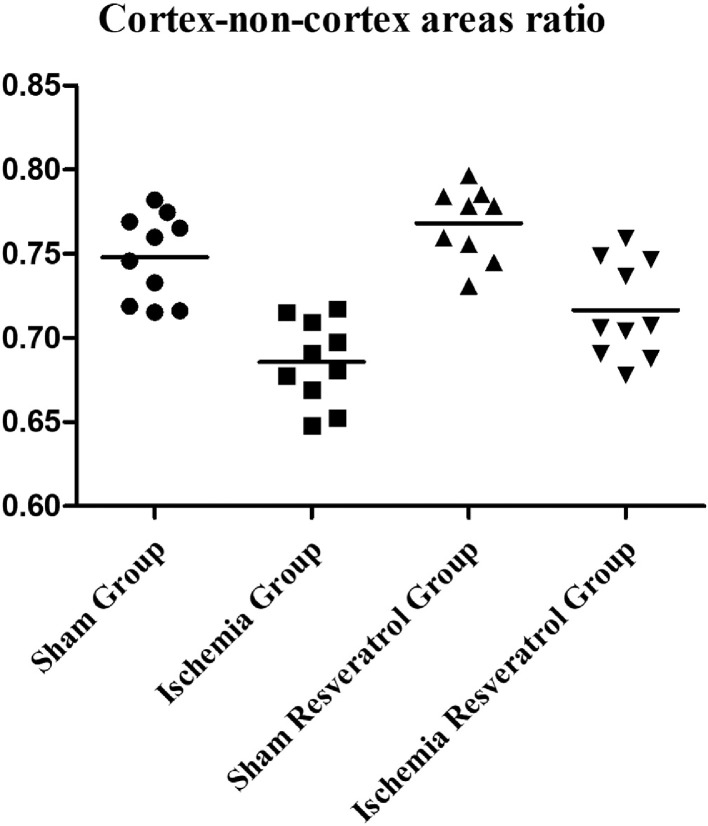
Cortex-non-cortex areas ration.

**Fig. 5:**
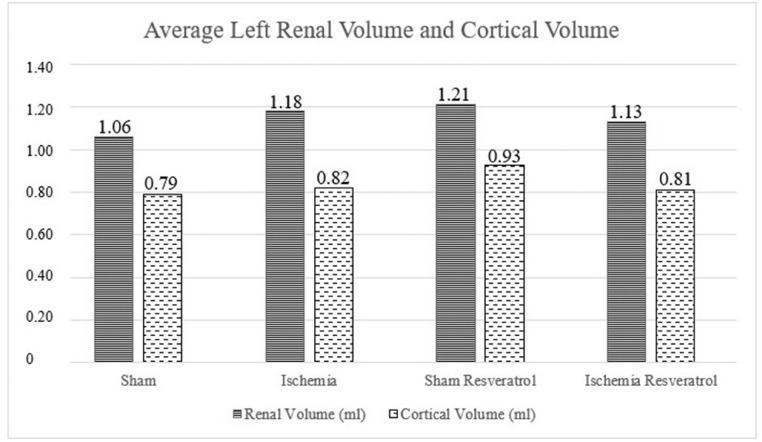
Average Left Renal Volume and Cortical Volume.

**Fig. 6:**
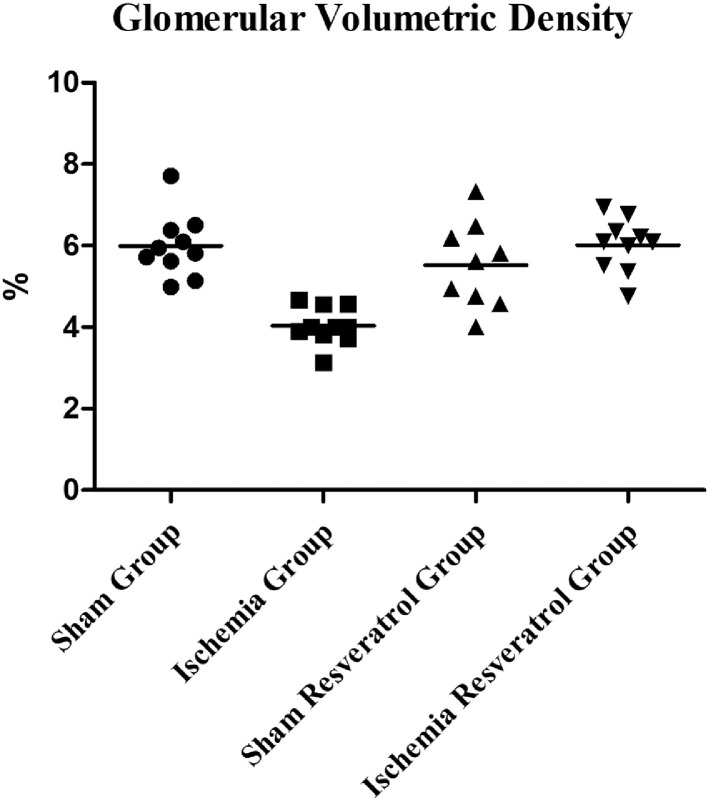
Glomerular Volumetric Density.

**Fig. 7:**
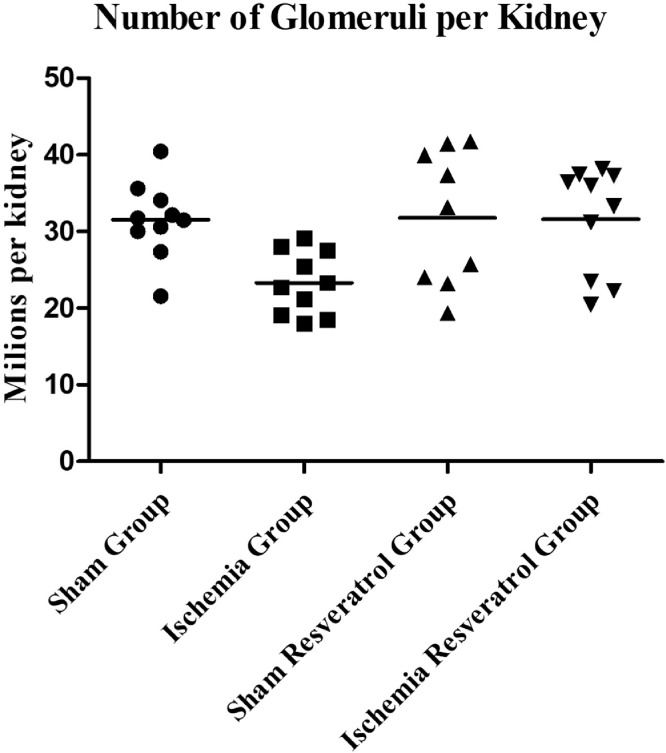
Number of Glomeruli per Kidney.

The present dataset describes the levels of serum biochemical markers, as well as morphometric and stereological analysis of rat kidneys submitted to 1-hour arteriovenous ischemia treated previously with resveratrol. Fig. 1 demonstrates the experimental and analytical steps that were conducted to obtain serum biochemical data for urea and creatinine. Fig. 2 demonstrates the experimental and analytical steps that were conducted to obtain the morphometric and stereological data, as well as their analysis. Fig. 3 describes the calculations to obtain specific data so that it was possible to calculate the N[G*lom*]. Fig. 4 scatter chart representing C—NC raw data from different groups’ animals; transversal black midline represents group's mean. Fig. 5 grouped column chart representing left renal volume and cortical volume averages of experimental groups; means are shown above the graph's bars. Fig. 6 scatter chart representing Vv[Glom] raw data from different groups’ animals; transversal black midline represents group's mean. Fig. 7 scatter chart representing N[Glom] raw data from different groups’ animals; transversal black midline represents group's mean. Table 1 shows raw data of serum urea and creatinine of rats submitted to sham surgery or to left renal warm ischemia with or without resveratrol treatment. Table 2 contains raw data of animals’ body weight, renal weight and volume of experimental groups. Table 3 includes raw data of kidney morphological data of rats submitted to sham surgery or to left renal warm ischemia with or without resveratrol treatment.

**Table 1:** Raw data: serum urea and creatinine of rats submitted to sham surgery or to left renal warm ischemia with or without resveratrol treatment.

Sham group	Serum urea (mg/dL)	Serum creatinine (mg/dL)	Ischemia group	Serum urea (mg/dL)	Serum creatinine (mg/dL)
Animal S1	42	0.46	Animal I1	43	0.42
Animal S2	41	0.44	Animal I2	50	0.44
Animal S3	40	0.44	Animal I3	44	0.45
Animal S4	42	0.45	Animal I4	40	0.51
Animal S5	41	0.42	Animal I5	43	0.50
Animal S6	43	0.45	Animal I6	50	0.51
Animal S7	39	0.45	Animal I7	51	0.46
Animal S8	41	0.42	Animal I8	45	0.50
Animal S9	45	0.44	Animal I9	44	0.45
Animal S10	41	0.45	Animal I10	48	0.50
Sham Resveratrol group	Serum urea (mg/dL)	Serum creatinine (mg/dL)	Ischemia Resveratrol group	Serum urea (mg/dL)	Serum creatinine (mg/dL)
Animal SR1	46	0.46	Animal IR1	49	0.46
Animal SR2	45	0.45	Animal IR2	42	0.53
Animal SR3	47	0.50	Animal IR3	43	0.49
Animal SR4	46	0.50	Animal IR4	43	0.51
Animal SR5	48	0.43	Animal IR5	45	0.43
Animal SR6	39	0.44	Animal IR6	42	0.50
Animal SR7	43	0.49	Animal IR7	44	0.49
Animal SR8	44	0.43	Animal IR8	42	0.44
Animal SR9	45	0.45	Animal IR9	40	0.39
			Animal IR10	43	0.40

**Table 2:** Raw data: body weight, renal weight and volume of experimental groups.

Animals from Sham group	Body weight (g)	Left kidney weight (g)	Left kidney volume (ml)	Right kidney weight (g)	Right kidney volume (ml)
Animal S1	298.0	0.93	0.91	1.08	1.04
Animal S2	301.0	1.05	1.01	1.11	1.08
Animal S3	278.5	1.00	0.98	1.09	1.05
Animal S4	291.5	1.01	1.00	1.01	0.99
Animal S5	368.5	1.11	1.07	1.13	1.10
Animal S6	299.0	0.90	0.88	0.95	0.93
Animal S7	329.5	1.04	1.00	0.97	0.93
Animal S8	357.5	1.20	1.17	1.13	1.11
Animal S9	369.0	1.17	1.19	1.28	1.29
Animal S10	346.5	1.41	1.37	1.32	1.31
Animals from Ischemia group	Body weight (g)	Left kidney weight (g)	Left kidney volume (ml)	Right kidney weight (g)	Right kidney volume (ml)
Animal I1	300.0	1.05	1.01	1.01	1.00
Animal I2	361.0	1.37	1.34	1.24	1.22
Animal I3	295.0	0.85	0.90	0.95	0.94
Animal I4	362.5	1.55	1.31	1.36	1.37
Animal I5	339.0	1.32	1.25	1.36	1.32
Animal I6	292.0	1.00	1.05	0.94	0.96
Animal I7	295.0	1.18	1.20	0.99	1.02
Animal I8	342.0	1.37	1.31	1.29	1.29
Animal I9	328.0	1.35	1.26	1.03	1.31
Animal I10	356.0	1.24	1.18	1.40	1.38
Animals from Sham Resveratrol group	Body weight (g)	Left kidney weight (g)	Left kidney volume (ml)	Right kidney weight (g)	Right kidney volume (ml)
Animal SR1	384.5	1.12	1.14	1.16	1.17
Animal SR2	376.0	1.14	1.15	1.25	1.23
Animal SR3	348.0	1.14	1.08	1.28	1.28
Animal SR4	353.0	1.12	1.05	1.11	1.09
Animal SR5	363.0	1.23	1.25	1.34	1.33
Animal SR6	298.0	1.03	1.04	1.12	1.11
Animal SR7	334.0	1.21	1.21	1.19	1.20
Animal SR8	348.5	1.50	1.50	1.37	1.37
Animal SR9	368.0	1.54	1.50	1.52	1.46
Animals from Ischemia Resveratrol group	Body weight (g)	Left kidney weight (g)	Left kidney volume (ml)	Right kidney weight (g)	Right kidney volume (ml)
Animal IR1	276.5	0.89	0.99	0.80	0.81
Animal IR2	369.5	1.12	1.12	1.11	1.22
Animal IR3	337.0	1.19	1.31	1.10	1.10
Animal IR4	302.5	1.04	1.04	0.98	0.98
Animal IR5	278.0	0.92	0.93	0.75	0.74
Animal IR6	323.5	1.31	1.31	1.16	1.17
Animal IR7	350.5	1.15	1.15	1.34	1.39
Animal IR8	331.0	1.27	1.23	1.17	1.13
Animal IR9	324.5	1.25	1.20	1.25	1.23
Animal IR10	290.5	1.05	0.99	1.05	0.98

**Table 3:** Raw data: Kidney morphological data of rats submitted to sham surgery or to left renal warm ischemia with or without resveratrol treatment.

Sham group	C-NC Ratio	Cortical Volume (ml)	Vv[Glom] (%)	Total glomerular volume (µm³)	VWGV (µm³)	N[Glom] (millions per kidney)
Animal S1	0.76912	0.69990	5.81428	40,694,579,756	1,887,222.214	21,563.21574
Animal S2	0.71523	0.72238	6.09523	44,031,304,147	1,388,244.613	31,717.25194
Animal S3	0.77467	0.75918	5.95238	45,189,604,684	1,477,171.314	30,591.98636
Animal S4	0.78191	0.78191	5.72380	44,755,106,953	1,635,263.227	27,368.74786
Animal S5	0.71621	0.76635	6.49947	49,808,765,114	1,659,965.089	30,005.91124
Animal S6	0.76534	0.67350	7.71428	51,955,834,587	1,284,496.795	40,448.39566
Animal S7	0.75988	0.75988	6.38095	48,488,087,942	1,422,827.219	34,078.69016
Animal S8	0.74567	0.87244	4.98842	43,521,230,900	1,353,662.007	32,150.73680
Animal S9	0.73288	0.87213	5.61904	49,005,799,925	1,556,217.271	31,490.33291
Animal S10	0.71888	0.98487	5.14285	50,650,821,126	1,422,827.219	35,598.71533
Ischemia group	C—NC Ratio	Cortical Volume (ml)	Vv[Glom] (%)	Total glomerular volume (µm³)	VWGV (µm³)	N[Glom] (millions per kidney)
Animal I1	0.69766	0.70463	4.56190	32,144,958,118	1,783,474.396	18,023.78447
Animal I2	0.69092	0.92583	4.00000	37,033,390,707	1,452,469.453	25,496.84652
Animal I3	0.71733	0.64560	4.00000	25,824,003,346	1,393,184.985	18,535.94722
Animal I4	0.67768	1.02330	3.90476	39,957,625,409	1,452,469.453	27,510.13134
Animal I5	0.65233	0.81541	4.57142	37,276,064,573	1,758,772.534	21,194.36359
Animal I6	0.71521	0.75097	3.71428	27,893,249,915	1,195,570.094	23,330.50154
Animal I7	0.70938	0.85126	4.00000	34,050,483,234	1,496,932.803	22,746.83484
Animal I8	0.66900	0.87639	3.80952	33,386,406,368	1,146,166.371	29,128.76108
Animal I9	0.647784	0.81620	3.14285	25,652,268,840	1,343,781.262	19,089.61641
Animal I10	0.68102	0.80361	4.66666	37,502,018,120	1,338,840.890	28,010.81025
Sham Resveratrol group	C-NC Ratio	Cortical volume (ml)	Vv[Glom] (%)	Total glomerular volume (µm³)	VWGV (µm³)	N[Glom] (millions per kidney)
Animal SR1	0.75965	0.86600	4.76190	41,238,541,789	1,778,534.024	23,186.81636
Animal SR2	0.73093	0.84057	4.00000	33,622,988,729	1,739,011.045	19,334.54582
Animal SR3	0.77838	0.84065	6.47619	54,442,400,557	1,363,542.751	39,927.16803
Animal SR4	0.75568	0.79346	4.95238	39,295,526,978	1,526,575.037	25,740.97311
Animal SR5	0.78412	0.98015	4.57142	44,806,879,708	1,862,520.352	24,057.12219
Animal SR6	0.79646	0.82832	7.33333	60,743,904,701	1,832,878.119	33,141.26787
Animal SR7	0.77845	0.94192	6.19047	58,309,860,659	1,561,157.643	37,350.39887
Animal SR8	0.78531	1.17796	5.61904	66,190,601,680	1,585,859.504	41,737.99854
Animal SR9	0.74494	1.11741	5.80952	64,916,243,536	1,566,098.015	41,450.94554
Ischemia Resveratrol group	C—NC Ratio	Cortical volume (ml)	Vv[Glom] (%)	Total glomerular volume (µm³)	VWGV (µm³)	N[Glom] (millions per kidney)
Animal IR1	0.75920	0.75161	6.95238	52,255,110,644	1,674,786.206	31,201.06344
Animal IR2	0.70738	0.79227	6.35238	50,328,361,859	1,343,781.262	37,452.79330
Animal IR3	0.68777	0.90098	6.20952	55,947,121,293	1,501,873.175	37,251.56172
Animal IR4	0.67783	0.70494	6.76190	47,667,778,483	1,324,019.773	36,002.31617
Animal IR5	0.70587	0.65646	6.09523	40,013,300,793	1,704,428.439	23,476.08141
Animal IR6	0.74879	0.98092	6.09523	59,789,779,915	1,640,203.600	36,452.65742
Animal IR7	0.74647	0.85844	5.52380	47,418,856,577	1,422,827.219	33,327.20653
Animal IR8	0.73676	0.90621	5.36666	48,633,737,173	1,274,616.050	38,155.59765
Animal IR9	0.69082	0.82899	4.76190	39,475,823,498	1,773,593.651	22,257.53541
Animal IR10	0.70410	0.69705	6.00000	41,823,540,000	2,045,314.127	20,448.46777

## 2 Experimental design, materials, and methods

All experiments were performed according to the national and international laws for scientific use of animals, and this project was formally approved by the local Ethics Committee for animal experimentation.

Male rats of Wistar lineage (*n* = 39; 9 weeks of age) were used, being allocated into 4 groups at random: Sham (S) – group submitted to open laparotomy and dissection of the renal pedicle; Sham Resveratrol (SR) – group previously treated with resveratrol and submitted to the same procedures of group Sham; Ischemia (I) - group submitted to 1-hour renal warm ischemia; Ischemia Resveratrol (IR) - group previously treated with resveratrol and submitted to 1-hour renal warm ischemia. Groups SR and IR received 30 mg/kg of resveratrol (Resveratrol, Terraternal, Santa Clara, USA) intraperitoneally 1 h before surgery, while untreated groups (S and I) received saline injections.

The animals were anesthetized via intramuscular ketamine (Cetamin, Syntec, Santana de Parnaíba, Brazil, 100 mg/kg) and xylazine (Xilazin, Syntec, 20 mg/kg). Once the surgical field was aseptic, a ventral midline incision was used to expose the abdominal viscera, which were displaced to expose the retroperitoneal area and the left kidney. The left renal artery and vein were isolated by blunt dissection. In rats of groups I and IR the renal vessels were clamped for 1 h, while in groups S and SR the pedicle was only dissected, and no ischemia was induced. All animals remained under anesthesia for 1 h, when the abdominal viscera were replaced, and the surgical wound was covered with moistened gauze. At the end of this period, vascular clamps were removed, and left kidney reperfusion was observed in groups I and IR. For all groups abdominal cavity was closed routinely.

The animals were killed a month after surgery by anesthetic overdose (Isoflurane, BioChimico, Rio de Janeiro, Brazil). A blood sample was collected by cardiac puncture during rats’ anesthetic plan, so it was possible to determine urea and creatinine serum levels by biochemical analysis (automated enzymatic method).

Both kidneys were dissected, collected and weighed. The renal volume was measured by the Scherle's method [1,2]. Left kidneys fixed in 4% buffered formaldehyde. The cortex-non-cortex areas ratio (C—NC) was achieved by morphometrical analysis of 2 mm transversal slices of left kidneys and calculated by the Cavalieri method [3], [4], [5]. The cortical volume (CV) was calculated by multiplying the renal volume by the C—NC [5].

Random samples from all 39 left kidneys were processed for paraffin embedding, sectioned at 5 µm thickness, and resulting histological blades were stained with haematoxylin and eosin. From each kidney, 25 histological fields, obtained from five different sections of the renal cortex, were photographed with a camera in a light microscope to be examined. Glomerular volumetric density (Vv[G*lom*]), which indicates the proportional volume occupied by the glomeruli in the cortex, was estimated by the point-counting method [3], [4], [5]. The volume-weighted mean glomerular volume (VWGV) was estimated by using the point-sampled intercepts method [3], [4], [5]. The estimation of the total number of glomeruli per kidney (N[G*lom*]) was achieved through the formula CVxVv[G*lom*]/VWGV [5].

Analyses were performed using GraphPad Prism 8.3.1 (GraphPad Software, San Diego, USA). The quantitative results were compared by one-way ANOVA with Tukey's post-test and all results were considered significant when the value of *p*<0.05.

## Declaration of Competing Interests

The authors declare that they have no known competing financial interests or personal relationships which have, or could be perceived to have, influenced the work reported in this article.

## Appendix Supplementary materials

Image, application 1

Image, application 2
