# Supplementary material for: Histomorphometric and biochemical data of rat kidney submitted to warm ischemia associated with resveratrol treatment
Source: Data Brief. 2020 Apr 15;30:105545. doi: 10.1016/j.dib.2020.105545 (PMC7186512; doi:10.1016/j.dib.2020.105545)
Supplement: Supplementary file 1 [file mmc1.docx]

0.93

0.82

1.21

1.18

1.40

1.20

1.00

0.80

0.60

0.40

0.20

0

1.13

0.81

0.79

1.06
